# Supplementary material for: Overlapping cell population expression profiling and regulatory inference in C. elegans
Source: BMC Genomics. 2016 Feb 29;17:159. doi: 10.1186/s12864-016-2482-z (PMC4772325; doi:10.1186/s12864-016-2482-z)
Supplement: Additional file 13: — Web supplement. (DOC 21 kb) [file 12864_2016_2482_MOESM13_ESM.zip › sortWeb/clusters/hier.300.clusters/50.html]

Cluster 50 

## Cluster 50

### Expression

| cnd-1 rep. 1 | cnd-1 rep. 2 | cnd-1 rep. 3 | pha-4 rep. 1 | pha-4 rep. 2 | pha-4 rep. 3 | ceh-27 | ceh-36 | ceh-6 | F21D5.9 | mir-57 | mls-2 | pal-1 | pros-1 | ttx-3 | unc-130 | hlh-16 | irx-1 | ceh-6 (+) hlh-16 (+) | ceh-6 (+) hlh-16 (-) | ceh-6 (-) hlh-16 (+) | cnd-1 singlets | pha-4 singlets | 0 | 60 | 120 | 150 | 180 | 240 | 330 | 390 | 420 | 480 | 540 | 570 | 600 | 630 | 660 | NAME | Functional description |
| --- | --- | --- | --- | --- | --- | --- | --- | --- | --- | --- | --- | --- | --- | --- | --- | --- | --- | --- | --- | --- | --- | --- | --- | --- | --- | --- | --- | --- | --- | --- | --- | --- | --- | --- | --- | --- | --- | --- | --- |
|  |  |  |  |  |  |  |  |  |  |  |  |  |  |  |  |  |  |  |  |  |  |  |  |  |  |  |  |  |  |  |  |  |  |  |  |  |  | K09C6.6 |  |
|  |  |  |  |  |  |  |  |  |  |  |  |  |  |  |  |  |  |  |  |  |  |  |  |  |  |  |  |  |  |  |  |  |  |  |  |  |  | T24D8.10 |  |
|  |  |  |  |  |  |  |  |  |  |  |  |  |  |  |  |  |  |  |  |  |  |  |  |  |  |  |  |  |  |  |  |  |  |  |  |  |  | F47F2.8 |  |
|  |  |  |  |  |  |  |  |  |  |  |  |  |  |  |  |  |  |  |  |  |  |  |  |  |  |  |  |  |  |  |  |  |  |  |  |  |  | Y48G8AL.17 |  |
|  |  |  |  |  |  |  |  |  |  |  |  |  |  |  |  |  |  |  |  |  |  |  |  |  |  |  |  |  |  |  |  |  |  |  |  |  |  | F10E9.19 |  |
|  |  |  |  |  |  |  |  |  |  |  |  |  |  |  |  |  |  |  |  |  |  |  |  |  |  |  |  |  |  |  |  |  |  |  |  |  |  | F47B7.13 |  |
|  |  |  |  |  |  |  |  |  |  |  |  |  |  |  |  |  |  |  |  |  |  |  |  |  |  |  |  |  |  |  |  |  |  |  |  |  |  | F35G12.14 |  |
|  |  |  |  |  |  |  |  |  |  |  |  |  |  |  |  |  |  |  |  |  |  |  |  |  |  |  |  |  |  |  |  |  |  |  |  |  |  | F35B12.15 |  |
|  |  |  |  |  |  |  |  |  |  |  |  |  |  |  |  |  |  |  |  |  |  |  |  |  |  |  |  |  |  |  |  |  |  |  |  |  |  | H24G06.2 |  |
|  |  |  |  |  |  |  |  |  |  |  |  |  |  |  |  |  |  |  |  |  |  |  |  |  |  |  |  |  |  |  |  |  |  |  |  |  |  | B0495.19 |  |
|  |  |  |  |  |  |  |  |  |  |  |  |  |  |  |  |  |  |  |  |  |  |  |  |  |  |  |  |  |  |  |  |  |  |  |  |  |  | Y41D4B.28 |  |
|  |  |  |  |  |  |  |  |  |  |  |  |  |  |  |  |  |  |  |  |  |  |  |  |  |  |  |  |  |  |  |  |  |  |  |  |  |  | T06A1.6 |  |
|  |  |  |  |  |  |  |  |  |  |  |  |  |  |  |  |  |  |  |  |  |  |  |  |  |  |  |  |  |  |  |  |  |  |  |  |  |  | C45E5.53 |  |
|  |  |  |  |  |  |  |  |  |  |  |  |  |  |  |  |  |  |  |  |  |  |  |  |  |  |  |  |  |  |  |  |  |  |  |  |  |  | C15C8.11 |  |
|  |  |  |  |  |  |  |  |  |  |  |  |  |  |  |  |  |  |  |  |  |  |  |  |  |  |  |  |  |  |  |  |  |  |  |  |  |  | F25F6.3 |  |
|  |  |  |  |  |  |  |  |  |  |  |  |  |  |  |  |  |  |  |  |  |  |  |  |  |  |  |  |  |  |  |  |  |  |  |  |  |  | F28A12.8 |  |
|  |  |  |  |  |  |  |  |  |  |  |  |  |  |  |  |  |  |  |  |  |  |  |  |  |  |  |  |  |  |  |  |  |  |  |  |  |  | C33G3.9 |  |
|  |  |  |  |  |  |  |  |  |  |  |  |  |  |  |  |  |  |  |  |  |  |  |  |  |  |  |  |  |  |  |  |  |  |  |  |  |  | F39D8.6 |  |
|  |  |  |  |  |  |  |  |  |  |  |  |  |  |  |  |  |  |  |  |  |  |  |  |  |  |  |  |  |  |  |  |  |  |  |  |  |  | C02B4.9 |  |
|  |  |  |  |  |  |  |  |  |  |  |  |  |  |  |  |  |  |  |  |  |  |  |  |  |  |  |  |  |  |  |  |  |  |  |  |  |  | R08B4.11 |  |
|  |  |  |  |  |  |  |  |  |  |  |  |  |  |  |  |  |  |  |  |  |  |  |  |  |  |  |  |  |  |  |  |  |  |  |  |  |  | T01C1.5 |  |
|  |  |  |  |  |  |  |  |  |  |  |  |  |  |  |  |  |  |  |  |  |  |  |  |  |  |  |  |  |  |  |  |  |  |  |  |  |  | B0403.8 |  |
|  |  |  |  |  |  |  |  |  |  |  |  |  |  |  |  |  |  |  |  |  |  |  |  |  |  |  |  |  |  |  |  |  |  |  |  |  |  | C42D8.14 |  |
|  |  |  |  |  |  |  |  |  |  |  |  |  |  |  |  |  |  |  |  |  |  |  |  |  |  |  |  |  |  |  |  |  |  |  |  |  |  | W01H2.9 |  |
|  |  |  |  |  |  |  |  |  |  |  |  |  |  |  |  |  |  |  |  |  |  |  |  |  |  |  |  |  |  |  |  |  |  |  |  |  |  | C04E7.t1 |  |
|  |  |  |  |  |  |  |  |  |  |  |  |  |  |  |  |  |  |  |  |  |  |  |  |  |  |  |  |  |  |  |  |  |  |  |  |  |  | *srw-38* | Serpentine Receptor, class W |
|  |  |  |  |  |  |  |  |  |  |  |  |  |  |  |  |  |  |  |  |  |  |  |  |  |  |  |  |  |  |  |  |  |  |  |  |  |  | Y75B12B.13 |  |
|  |  |  |  |  |  |  |  |  |  |  |  |  |  |  |  |  |  |  |  |  |  |  |  |  |  |  |  |  |  |  |  |  |  |  |  |  |  | E02A10.6 |  |
|  |  |  |  |  |  |  |  |  |  |  |  |  |  |  |  |  |  |  |  |  |  |  |  |  |  |  |  |  |  |  |  |  |  |  |  |  |  | C05A2.2 |  |
|  |  |  |  |  |  |  |  |  |  |  |  |  |  |  |  |  |  |  |  |  |  |  |  |  |  |  |  |  |  |  |  |  |  |  |  |  |  | K08B12.7 |  |
|  |  |  |  |  |  |  |  |  |  |  |  |  |  |  |  |  |  |  |  |  |  |  |  |  |  |  |  |  |  |  |  |  |  |  |  |  |  | K02H11.8 |  |
|  |  |  |  |  |  |  |  |  |  |  |  |  |  |  |  |  |  |  |  |  |  |  |  |  |  |  |  |  |  |  |  |  |  |  |  |  |  | K04D7.13 |  |
|  |  |  |  |  |  |  |  |  |  |  |  |  |  |  |  |  |  |  |  |  |  |  |  |  |  |  |  |  |  |  |  |  |  |  |  |  |  | *his-34* | HIStone |
|  |  |  |  |  |  |  |  |  |  |  |  |  |  |  |  |  |  |  |  |  |  |  |  |  |  |  |  |  |  |  |  |  |  |  |  |  |  | F54C8.10 |  |
|  |  |  |  |  |  |  |  |  |  |  |  |  |  |  |  |  |  |  |  |  |  |  |  |  |  |  |  |  |  |  |  |  |  |  |  |  |  | Y42G9A.8 |  |
|  |  |  |  |  |  |  |  |  |  |  |  |  |  |  |  |  |  |  |  |  |  |  |  |  |  |  |  |  |  |  |  |  |  |  |  |  |  | B0244.t2 |  |
|  |  |  |  |  |  |  |  |  |  |  |  |  |  |  |  |  |  |  |  |  |  |  |  |  |  |  |  |  |  |  |  |  |  |  |  |  |  | E04D5.8 |  |
|  |  |  |  |  |  |  |  |  |  |  |  |  |  |  |  |  |  |  |  |  |  |  |  |  |  |  |  |  |  |  |  |  |  |  |  |  |  | *chil-17* | CHItinase-Like |
|  |  |  |  |  |  |  |  |  |  |  |  |  |  |  |  |  |  |  |  |  |  |  |  |  |  |  |  |  |  |  |  |  |  |  |  |  |  | C28F5.6 |  |
|  |  |  |  |  |  |  |  |  |  |  |  |  |  |  |  |  |  |  |  |  |  |  |  |  |  |  |  |  |  |  |  |  |  |  |  |  |  | T25D10.6 |  |
|  |  |  |  |  |  |  |  |  |  |  |  |  |  |  |  |  |  |  |  |  |  |  |  |  |  |  |  |  |  |  |  |  |  |  |  |  |  | C33F10.16 |  |
|  |  |  |  |  |  |  |  |  |  |  |  |  |  |  |  |  |  |  |  |  |  |  |  |  |  |  |  |  |  |  |  |  |  |  |  |  |  | *fbxa-165* | F-box A protein |
|  |  |  |  |  |  |  |  |  |  |  |  |  |  |  |  |  |  |  |  |  |  |  |  |  |  |  |  |  |  |  |  |  |  |  |  |  |  | Y51H7BR.11 |  |
|  |  |  |  |  |  |  |  |  |  |  |  |  |  |  |  |  |  |  |  |  |  |  |  |  |  |  |  |  |  |  |  |  |  |  |  |  |  | F09C3.9 |  |
|  |  |  |  |  |  |  |  |  |  |  |  |  |  |  |  |  |  |  |  |  |  |  |  |  |  |  |  |  |  |  |  |  |  |  |  |  |  | Y71A12B.29 |  |
|  |  |  |  |  |  |  |  |  |  |  |  |  |  |  |  |  |  |  |  |  |  |  |  |  |  |  |  |  |  |  |  |  |  |  |  |  |  | F58D5.8 |  |
|  |  |  |  |  |  |  |  |  |  |  |  |  |  |  |  |  |  |  |  |  |  |  |  |  |  |  |  |  |  |  |  |  |  |  |  |  |  | C17D12.12 |  |
|  |  |  |  |  |  |  |  |  |  |  |  |  |  |  |  |  |  |  |  |  |  |  |  |  |  |  |  |  |  |  |  |  |  |  |  |  |  | K02F2.9 |  |
|  |  |  |  |  |  |  |  |  |  |  |  |  |  |  |  |  |  |  |  |  |  |  |  |  |  |  |  |  |  |  |  |  |  |  |  |  |  | Y39G10AR.26 |  |
|  |  |  |  |  |  |  |  |  |  |  |  |  |  |  |  |  |  |  |  |  |  |  |  |  |  |  |  |  |  |  |  |  |  |  |  |  |  | Y54E10A.13 |  |
|  |  |  |  |  |  |  |  |  |  |  |  |  |  |  |  |  |  |  |  |  |  |  |  |  |  |  |  |  |  |  |  |  |  |  |  |  |  | F46F2.10 |  |
|  |  |  |  |  |  |  |  |  |  |  |  |  |  |  |  |  |  |  |  |  |  |  |  |  |  |  |  |  |  |  |  |  |  |  |  |  |  | F55E10.8 |  |
|  |  |  |  |  |  |  |  |  |  |  |  |  |  |  |  |  |  |  |  |  |  |  |  |  |  |  |  |  |  |  |  |  |  |  |  |  |  | W06B11.6 |  |
|  |  |  |  |  |  |  |  |  |  |  |  |  |  |  |  |  |  |  |  |  |  |  |  |  |  |  |  |  |  |  |  |  |  |  |  |  |  | T07F12.7 |  |
|  |  |  |  |  |  |  |  |  |  |  |  |  |  |  |  |  |  |  |  |  |  |  |  |  |  |  |  |  |  |  |  |  |  |  |  |  |  | Y80D3A.15 |  |
|  |  |  |  |  |  |  |  |  |  |  |  |  |  |  |  |  |  |  |  |  |  |  |  |  |  |  |  |  |  |  |  |  |  |  |  |  |  | *srz-35* | Serpentine Receptor, class Z |
|  |  |  |  |  |  |  |  |  |  |  |  |  |  |  |  |  |  |  |  |  |  |  |  |  |  |  |  |  |  |  |  |  |  |  |  |  |  | *scrm-8* | SCRaMblase (phospholipid scramblase) |
|  |  |  |  |  |  |  |  |  |  |  |  |  |  |  |  |  |  |  |  |  |  |  |  |  |  |  |  |  |  |  |  |  |  |  |  |  |  | T28D6.t2 |  |
|  |  |  |  |  |  |  |  |  |  |  |  |  |  |  |  |  |  |  |  |  |  |  |  |  |  |  |  |  |  |  |  |  |  |  |  |  |  | T21C12.10 |  |
|  |  |  |  |  |  |  |  |  |  |  |  |  |  |  |  |  |  |  |  |  |  |  |  |  |  |  |  |  |  |  |  |  |  |  |  |  |  | *clec-158* | C-type LECtin |
|  |  |  |  |  |  |  |  |  |  |  |  |  |  |  |  |  |  |  |  |  |  |  |  |  |  |  |  |  |  |  |  |  |  |  |  |  |  | C36F7.24 |  |
|  |  |  |  |  |  |  |  |  |  |  |  |  |  |  |  |  |  |  |  |  |  |  |  |  |  |  |  |  |  |  |  |  |  |  |  |  |  | R06B10.7 |  |
|  |  |  |  |  |  |  |  |  |  |  |  |  |  |  |  |  |  |  |  |  |  |  |  |  |  |  |  |  |  |  |  |  |  |  |  |  |  | C18F3.9 |  |
|  |  |  |  |  |  |  |  |  |  |  |  |  |  |  |  |  |  |  |  |  |  |  |  |  |  |  |  |  |  |  |  |  |  |  |  |  |  | F27E11.6 |  |
|  |  |  |  |  |  |  |  |  |  |  |  |  |  |  |  |  |  |  |  |  |  |  |  |  |  |  |  |  |  |  |  |  |  |  |  |  |  | C14F11.10 |  |
|  |  |  |  |  |  |  |  |  |  |  |  |  |  |  |  |  |  |  |  |  |  |  |  |  |  |  |  |  |  |  |  |  |  |  |  |  |  | F33D11.14 |  |
|  |  |  |  |  |  |  |  |  |  |  |  |  |  |  |  |  |  |  |  |  |  |  |  |  |  |  |  |  |  |  |  |  |  |  |  |  |  | F52E10.10 |  |
|  |  |  |  |  |  |  |  |  |  |  |  |  |  |  |  |  |  |  |  |  |  |  |  |  |  |  |  |  |  |  |  |  |  |  |  |  |  | C31H2.5 |  |
|  |  |  |  |  |  |  |  |  |  |  |  |  |  |  |  |  |  |  |  |  |  |  |  |  |  |  |  |  |  |  |  |  |  |  |  |  |  | K10C3.12 |  |
|  |  |  |  |  |  |  |  |  |  |  |  |  |  |  |  |  |  |  |  |  |  |  |  |  |  |  |  |  |  |  |  |  |  |  |  |  |  | F59D12.8 |  |
|  |  |  |  |  |  |  |  |  |  |  |  |  |  |  |  |  |  |  |  |  |  |  |  |  |  |  |  |  |  |  |  |  |  |  |  |  |  | *srh-5* | Serpentine Receptor, class H |
|  |  |  |  |  |  |  |  |  |  |  |  |  |  |  |  |  |  |  |  |  |  |  |  |  |  |  |  |  |  |  |  |  |  |  |  |  |  | F09G2.13 |  |
|  |  |  |  |  |  |  |  |  |  |  |  |  |  |  |  |  |  |  |  |  |  |  |  |  |  |  |  |  |  |  |  |  |  |  |  |  |  | *clec-94* | C-type LECtin |
|  |  |  |  |  |  |  |  |  |  |  |  |  |  |  |  |  |  |  |  |  |  |  |  |  |  |  |  |  |  |  |  |  |  |  |  |  |  | ZK1251.15 |  |
|  |  |  |  |  |  |  |  |  |  |  |  |  |  |  |  |  |  |  |  |  |  |  |  |  |  |  |  |  |  |  |  |  |  |  |  |  |  | C18H7.12 |  |
|  |  |  |  |  |  |  |  |  |  |  |  |  |  |  |  |  |  |  |  |  |  |  |  |  |  |  |  |  |  |  |  |  |  |  |  |  |  | *msp-10* | Major Sperm Protein |
|  |  |  |  |  |  |  |  |  |  |  |  |  |  |  |  |  |  |  |  |  |  |  |  |  |  |  |  |  |  |  |  |  |  |  |  |  |  | R06C1.14 |  |
|  |  |  |  |  |  |  |  |  |  |  |  |  |  |  |  |  |  |  |  |  |  |  |  |  |  |  |  |  |  |  |  |  |  |  |  |  |  | C14F11.19 |  |
|  |  |  |  |  |  |  |  |  |  |  |  |  |  |  |  |  |  |  |  |  |  |  |  |  |  |  |  |  |  |  |  |  |  |  |  |  |  | *srh-80* | Serpentine Receptor, class H |
|  |  |  |  |  |  |  |  |  |  |  |  |  |  |  |  |  |  |  |  |  |  |  |  |  |  |  |  |  |  |  |  |  |  |  |  |  |  | *col-161* | COLlagen |
|  |  |  |  |  |  |  |  |  |  |  |  |  |  |  |  |  |  |  |  |  |  |  |  |  |  |  |  |  |  |  |  |  |  |  |  |  |  | *tbx-37* | T BoX family |
|  |  |  |  |  |  |  |  |  |  |  |  |  |  |  |  |  |  |  |  |  |  |  |  |  |  |  |  |  |  |  |  |  |  |  |  |  |  | F35G2.9 |  |
|  |  |  |  |  |  |  |  |  |  |  |  |  |  |  |  |  |  |  |  |  |  |  |  |  |  |  |  |  |  |  |  |  |  |  |  |  |  | F26F12.18 |  |
|  |  |  |  |  |  |  |  |  |  |  |  |  |  |  |  |  |  |  |  |  |  |  |  |  |  |  |  |  |  |  |  |  |  |  |  |  |  | T09B9.8 |  |
|  |  |  |  |  |  |  |  |  |  |  |  |  |  |  |  |  |  |  |  |  |  |  |  |  |  |  |  |  |  |  |  |  |  |  |  |  |  | B0350.82 |  |
|  |  |  |  |  |  |  |  |  |  |  |  |  |  |  |  |  |  |  |  |  |  |  |  |  |  |  |  |  |  |  |  |  |  |  |  |  |  | C39D10.16 |  |
|  |  |  |  |  |  |  |  |  |  |  |  |  |  |  |  |  |  |  |  |  |  |  |  |  |  |  |  |  |  |  |  |  |  |  |  |  |  | Y46B2A.2 |  |
|  |  |  |  |  |  |  |  |  |  |  |  |  |  |  |  |  |  |  |  |  |  |  |  |  |  |  |  |  |  |  |  |  |  |  |  |  |  | *anr-15* | Antisense Non-coding RNA |
|  |  |  |  |  |  |  |  |  |  |  |  |  |  |  |  |  |  |  |  |  |  |  |  |  |  |  |  |  |  |  |  |  |  |  |  |  |  | T22D1.1 |  |
|  |  |  |  |  |  |  |  |  |  |  |  |  |  |  |  |  |  |  |  |  |  |  |  |  |  |  |  |  |  |  |  |  |  |  |  |  |  | F40F9.13 |  |
|  |  |  |  |  |  |  |  |  |  |  |  |  |  |  |  |  |  |  |  |  |  |  |  |  |  |  |  |  |  |  |  |  |  |  |  |  |  | K08D8.11 |  |
|  |  |  |  |  |  |  |  |  |  |  |  |  |  |  |  |  |  |  |  |  |  |  |  |  |  |  |  |  |  |  |  |  |  |  |  |  |  | T19C4.t1 |  |
|  |  |  |  |  |  |  |  |  |  |  |  |  |  |  |  |  |  |  |  |  |  |  |  |  |  |  |  |  |  |  |  |  |  |  |  |  |  | F43C11.13 |  |
|  |  |  |  |  |  |  |  |  |  |  |  |  |  |  |  |  |  |  |  |  |  |  |  |  |  |  |  |  |  |  |  |  |  |  |  |  |  | C08B6.16 |  |
|  |  |  |  |  |  |  |  |  |  |  |  |  |  |  |  |  |  |  |  |  |  |  |  |  |  |  |  |  |  |  |  |  |  |  |  |  |  | H30A04.11 |  |
|  |  |  |  |  |  |  |  |  |  |  |  |  |  |  |  |  |  |  |  |  |  |  |  |  |  |  |  |  |  |  |  |  |  |  |  |  |  | *srg-11* | Serpentine Receptor, class G (gamma) |
|  |  |  |  |  |  |  |  |  |  |  |  |  |  |  |  |  |  |  |  |  |  |  |  |  |  |  |  |  |  |  |  |  |  |  |  |  |  | R11B5.3 |  |

### Phenotypes enriched

none found

### Anatomy terms enriched

none found

### GO terms enriched

none found

### Expression clusters enriched

none found

### Motifs enriched

|  |  |  |  |  |  |
| --- | --- | --- | --- | --- | --- |
| **Motif** | **Logo** | **Possible orthologs** | **Number of motifs in cluster** | **Enrichment** | **FDR corrected p** |
| pTH9135 |  | pop-1 | 56 | 1.87 | 1.5e-05 |
| MA0495.1 |  | crh-1 F45H11.6 | 45 | 1.94 | 2.8e-04 |
| MA0468.1 |  | alr-1 ZC204.2 | 38 | 2.02 | 9.4e-04 |
| KLF1\_f1 |  | klf-1 | 28 | 2.43 | 9.7e-04 |
| MEIS1\_1 |  | ceh-32 ceh-20 | 48 | 1.76 | 1.2e-03 |
| pTH9082 |  | mab-23 | 33 | 2.16 | 1.3e-03 |
| pTH10816 |  | dmd-6 | 49 | 1.73 | 1.5e-03 |
| NKX21\_f1 |  | ceh-22 dsc-1 | 16 | 3.59 | 1.5e-03 |
| CREB1\_f1 |  | atf-5 crh-1 | 41 | 1.86 | 2.2e-03 |
| pTH10721 |  | ztf-9 ceh-18 | 49 | 1.69 | 2.4e-03 |
| COT2\_f1 |  | nhr-2 | 55 | 1.60 | 2.5e-03 |
| EGR2\_si |  | ZC328.2 | 16 | 3.34 | 3.1e-03 |
| HXA7\_f1 |  | lin-39 | 36 | 1.93 | 4.1e-03 |
| N$SKN1\_01 |  | ceh-2 skn-1 | 27 | 2.25 | 4.4e-03 |
| PAX6\_f1 |  | pax-3 (-0.51) pax-2 | 13 | 3.84 | 4.4e-03 |
| pTH9314 |  | atf-7 | 37 | 1.89 | 4.4e-03 |
| Nkx2-9\_3082 |  | ceh-24 dsc-1 | 18 | 2.93 | 4.5e-03 |
| pTH9925 |  | ztf-11 | 65 | 1.44 | 4.5e-03 |
| TBX20\_2 |  | tbx-39 mab-9 | 16 | 3.16 | 5.2e-03 |
| Pbx1\_3203 |  | ceh-20 | 24 | 2.38 | 5.4e-03 |
| Irx3\_0920 |  | irx-1 | 48 | 1.64 | 6.2e-03 |
| V$DELTAEF1\_01 |  | hlh-2 ztf-6 | 42 | 1.74 | 6.6e-03 |
| pTH6327 |  | dsc-1 | 28 | 2.13 | 7.3e-03 |
| nau\_da\_SANGER\_5\_FBgn0002922 |  | ces-1 hlh-15 hlh-1 | 47 | 1.64 | 7.6e-03 |
| pTH2820 |  | ZC328.2 | 14 | 3.36 | 7.7e-03 |
| pTH10795 |  | ztf-14 | 42 | 1.71 | 9.0e-03 |
| FOXO1\_3 |  | daf-16 ZC328.2 | 49 | 1.59 | 9.7e-03 |
| TBX3\_f1 |  | tbx-39 | 28 | 2.06 | 1.1e-02 |
| Hey\_SANGER\_5\_FBgn0027788 |  | lin-22 | 17 | 2.77 | 1.2e-02 |
| pTH9108 |  | daf-12 | 20 | 2.48 | 1.2e-02 |
| V$YY1\_01 |  | lsy-2 | 17 | 2.74 | 1.3e-02 |
| pTH10788 |  | tbx-33 | 44 | 1.64 | 1.3e-02 |
| Mv75 |  | elt-1 | 31 | 1.93 | 1.3e-02 |
| MA0085.1 |  | lag-1 | 38 | 1.75 | 1.4e-02 |
| COT2\_f2 |  | nhr-2 | 54 | 1.50 | 1.4e-02 |
| MAFA\_f1 |  | F45H11.6 | 34 | 1.84 | 1.5e-02 |
| MA0261.1 |  | lin-14 | 41 | 1.68 | 1.5e-02 |
| Mw151 |  | gei-11 | 36 | 1.78 | 1.6e-02 |
| Irx3\_2226 |  | irx-1 | 48 | 1.57 | 1.6e-02 |
| pTH9915 |  | zip-3 | 40 | 1.69 | 1.6e-02 |
| HXB8\_do |  | lin-39 | 19 | 2.48 | 1.7e-02 |
| THRB\_3 |  | nhr-15 | 26 | 2.06 | 1.8e-02 |
| V$GATA1\_01 |  | elt-1 | 28 | 1.99 | 1.8e-02 |
| Spt15 |  | tbp-1 | 25 | 2.10 | 1.8e-02 |
| Eip74EF\_FlyReg\_FBgn0000567 |  | C24A1.2 | 45 | 1.60 | 1.9e-02 |
| Sox1\_2 |  | sox-4 | 32 | 1.85 | 2.0e-02 |
| POU3F2\_2 |  | sox-4 ceh-18 | 41 | 1.65 | 2.1e-02 |
| Barhl1\_1 |  | ceh-31 | 9 | 4.29 | 2.2e-02 |
| V$GATA1\_03 |  | elt-1 | 26 | 2.03 | 2.2e-02 |
| pTH10040 |  | slr-2 | 44 | 1.60 | 2.2e-02 |
| MA0165.1 |  | lin-39 ceh-24 | 57 | 1.44 | 2.3e-02 |
| MEIS2\_do |  | ceh-32 | 41 | 1.64 | 2.3e-02 |
| MA0452.2 |  | B0310.2 | 11 | 3.52 | 2.4e-02 |
| Tcf3\_3787 |  | pop-1 | 46 | 1.56 | 2.4e-02 |
| pTH9182 |  | tbx-39 | 37 | 1.71 | 2.5e-02 |
| SOX10\_2 |  | sox-4 | 20 | 2.31 | 2.5e-02 |
| pTH2283 |  | odd-2 | 44 | 1.59 | 2.5e-02 |
| pTH6425 |  | ceh-20 | 13 | 3.05 | 2.5e-02 |
| Rfx4\_3761 |  | daf-19 | 30 | 1.87 | 2.5e-02 |
| Mafk\_3106 |  | F45H11.6 | 43 | 1.60 | 2.6e-02 |
| V$CDPCR1\_01 |  | ceh-48 | 18 | 2.45 | 2.6e-02 |
| pTH10777 |  | dmd-3 | 29 | 1.90 | 2.6e-02 |
| V$AREB6\_01 |  | ztf-6 | 10 | 3.75 | 2.6e-02 |
| Mw142 |  | egl-27 elt-1 | 52 | 1.48 | 2.6e-02 |
| pTH5781 |  | ceh-32 | 12 | 3.20 | 2.8e-02 |
| pTH9237 |  | mel-28 | 56 | 1.43 | 2.9e-02 |
| Sox1\_4 |  | sox-4 | 31 | 1.83 | 2.9e-02 |
| MA0079.3 |  | ZC328.2 klf-2 | 48 | 1.52 | 3.0e-02 |
| pTH9049 |  | ztf-6 ztf-2 | 52 | 1.47 | 3.0e-02 |
| Irx5\_2385 |  | irx-1 | 42 | 1.60 | 3.0e-02 |
| TFEB\_f1 |  | aha-1 hlh-30 | 26 | 1.97 | 3.1e-02 |
| pTH9244 |  | tbx-39 | 37 | 1.67 | 3.5e-02 |
| I$ABDB\_01 |  | ceh-24 | 47 | 1.52 | 3.5e-02 |
| Nkx6-3\_3446 |  | cog-1 | 33 | 1.75 | 3.5e-02 |
| ETV4\_f1 |  | lin-1 | 49 | 1.49 | 3.6e-02 |
| pTH10796 |  | hsf-1 | 39 | 1.63 | 3.7e-02 |
| V$VMYB\_01 |  | D1081.8 | 55 | 1.43 | 3.7e-02 |
| MA0594.1 |  | lin-39 | 14 | 2.72 | 3.9e-02 |
| V$PAX2\_02 |  | pax-1 | 46 | 1.52 | 4.0e-02 |
| pTH9913 |  | skn-1 | 41 | 1.59 | 4.1e-02 |
| MA0126.1 |  | pax-3 (-0.51) lin-48 | 51 | 1.46 | 4.1e-02 |
| pTH10623 |  | scrt-1 | 29 | 1.83 | 4.2e-02 |
| Hoxc10\_2779 |  | php-3 | 7 | 4.82 | 4.3e-02 |
| pTH6508 |  | nhr-36 | 33 | 1.73 | 4.3e-02 |
| pTH9884 |  | tbx-39 | 21 | 2.12 | 4.5e-02 |
| Oli\_da\_SANGER\_5\_3\_FBgn0032651 |  | hlh-32 | 37 | 1.64 | 4.5e-02 |
| Ptx1\_SOLEXA\_FBgn0020912 |  | alr-1 | 28 | 1.84 | 4.6e-02 |
| So\_Cell\_FBgn0003460 |  | ceh-32 | 30 | 1.78 | 4.7e-02 |
| pTH9916 |  | W08E12.1 | 29 | 1.81 | 4.7e-02 |
| V$POU3F2\_01 |  | ceh-18 | 39 | 1.60 | 4.7e-02 |
| pTH1292 |  | pzf-1 | 47 | 1.49 | 4.9e-02 |
| PURA\_f1 |  | plp-2 | 33 | 1.71 | 4.9e-02 |
| Sox4 |  | pop-1 sox-4 | 58 | 1.38 | 4.9e-02 |
| V$AREB6\_02 |  | ztf-6 | 21 | 2.09 | 5.0e-02 |

### Correlated (and anti-correlated) transcription factors

|  |  |
| --- | --- |
| **Transcription factor** | **Correlation** |
| tbx-37 | 0.64 |
| daf-8 | 0.54 |
| nhr-72 | 0.47 |
| T07D10.3 | 0.44 |
| cfi-1 | 0.42 |
| Y53F4B.5 | 0.42 |
| hlh-14 | 0.39 |
| grl-25 | 0.39 |
| C46E10.9 | 0.39 |
| F38C2.7 | 0.39 |
| Y54G2A.20 | 0.39 |
| elt-6 | 0.39 |
| Y53G8AR.9 | 0.39 |
| atf-2 | 0.37 |
| odr-7 | 0.37 |
| nfya-1 | 0.37 |
| nhr-216 | 0.36 |
| nhr-173 | 0.36 |
| egl-43 | 0.36 |
| ngn-1 | 0.35 |
| ceh-39 | 0.35 |
| nhr-156 | 0.35 |
| tab-1 | 0.35 |
| efl-2 | 0.35 |
| B0250.4 | 0.35 |
| nhr-68 | -0.38 |
| T26A5.8 | -0.38 |
| nhr-80 | -0.38 |
| nhr-114 | -0.38 |
| elk-2 | -0.39 |
| nhr-176 | -0.40 |
| ets-8 | -0.41 |
| pos-1 | -0.41 |
| nhr-149 | -0.43 |
| mbf-1 | -0.43 |
| nhr-210 | -0.44 |
| nhr-245 | -0.46 |
| nhr-109 | -0.46 |
| nhr-43 | -0.47 |
| nhr-218 | -0.48 |
| end-1 | -0.48 |
| nhr-270 | -0.48 |
| cey-2 | -0.49 |
| nhr-81 | -0.49 |
| tbx-8 | -0.50 |
| pax-3 | -0.51 |
| nhr-150 | -0.51 |
| hsf-2 | -0.54 |
| nhr-127 | -0.55 |
| nhr-171 | -0.57 |

### ChIP peaks enriched

none found
